# Distillation of the clinical algorithm improves prognosis by multi-task deep learning in high-risk Neuroblastoma

**DOI:** 10.1371/journal.pone.0208924

**Published:** 2018-12-07

**Authors:** Valerio Maggio, Marco Chierici, Giuseppe Jurman, Cesare Furlanello

**Affiliations:** Fondazione Bruno Kessler, Trento, Italy; National Taiwan University, TAIWAN

## Abstract

We introduce the CDRP (Concatenated Diagnostic-Relapse Prognostic) architecture for multi-task deep learning that incorporates a clinical algorithm, *e*.*g*., a risk stratification schema to improve prognostic profiling. We present the first application to survival prediction in High-Risk (HR) Neuroblastoma from transcriptomics data, a task that studies from the MAQC consortium have shown to remain the hardest among multiple diagnostic and prognostic endpoints predictable from the same dataset. To obtain a more accurate risk stratification needed for appropriate treatment strategies, CDRP combines a first component (CDRP-A) synthesizing a diagnostic task and a second component (CDRP-N) dedicated to one or more prognostic tasks. The approach leverages the advent of semi-supervised deep learning structures that can flexibly integrate multimodal data or internally create multiple processing paths. CDRP-A is an autoencoder trained on gene expression on the HR/non-HR risk stratification by the Children’s Oncology Group, obtaining a 64-node representation in the bottleneck layer. CDRP-N is a multi-task classifier for two prognostic endpoints, *i*.*e*., Event-Free Survival (EFS) and Overall Survival (OS). CDRP-A provides the HR embedding input to the CDRP-N shared layer, from which two branches depart to model EFS and OS, respectively. To control for selection bias, CDRP is trained and evaluated using a Data Analysis Protocol (DAP) developed within the MAQC initiative. CDRP was applied on Illumina RNA-Seq of 498 Neuroblastoma patients (HR: 176) from the SEQC study (12,464 Entrez genes) and on Affymetrix Human Exon Array expression profiles (17,450 genes) of 247 primary diagnostic Neuroblastoma of the TARGET NBL cohort. On the SEQC HR patients, CDRP achieves Matthews Correlation Coefficient (MCC) 0.38 for EFS and MCC = 0.19 for OS in external validation, improving over published SEQC models. We show that a CDRP-N embedding is indeed parametrically associated to increasing severity and the embedding can be used to better stratify patients’ survival.

## Introduction

The challenge of dealing with multiple endpoints of clinical interest is a key challenge of predictive models from high-throughput omics data, as found in the MAQC-II (Microarray Analysis and Quality Control) study [1]. Neuroblastoma is a paradigmatic example of disease where the medical community has adopted a clinical algorithm to assign risk status. Severity of cancer and therapeutic options are computed as a combination of clinical information and specific biomarkers. However, the precision medicine approach aims at identifying more accurately the subtypes of patients in terms of expected response to therapy. In Neuroblastoma, high throughput molecular profiling still fails to identify molecular profiles clearly associated to high risk (HR) subtypes, for which successful therapy cannot be warranted yet. Arising predominantly in the first two years of life, Neuroblastoma is the most frequent extracranial solid tumor in infancy, accounting for about 500 new cases in Europe per year (130 in Germany), corresponding to roughly 8% of pediatric cancers and 15% of pediatric oncology deaths [2].

Neuroblastoma develops from the immature cells of the ganglionic sympathetic nervous system lineage stemming from the neural crest cells, and tumors can arise at any site where sympathetic neuroblasts are present during normal development [3], *e*.*g*., in chest. The broad variety of clinical behavior represents Neuroblastoma’s major hallmark, ranging from spontaneous regression (stage 4S) to gradual maturation (stages 1 − 2) to aggressive and often fatal ganglioneuroma [4, 5] (stages 3 − 4), despite intensive multimodal treatment. Official staging is defined by the International Neuroblastoma Staging System (INSS) [6]. The current strategies for designing tumor treatment therapies use different combinations of clinical and genetic markers to discriminate patients with low or high risk of death from the disease. The features used for this decision include age [7], tumor stage [8, 9] and MYCN proto-oncogene genomic amplification [10, 11]. However, this standard protocol is still imperfect, often resulting in over- or under-treatment of patients with Neuroblastoma [12]. Cancer genetic instability is most often studied at the genomic and gene expression levels, focusing on the effects of genomic alterations on transcription and splicing. In fact, several studies demonstrated that using messenger RNA (mRNA) expression information for molecular classification improves the diagnostic accuracy over traditional clinical markers for individual tumor behavior, enhancing the risk stratification reliability and therefore the therapy selection [1, 13–19]. Only a limited number of the published classifiers based on gene expression have been so far incorporated into clinical operative systems for a controlled validation trial: as examples, [20, 21] and the U.S. National Institutes of Health clinical trials [22, 23]. The reasons are diverse and include logistic or bureaucratic hindrances for the implementation of classifiers into clinical practice, difficulties in the setup of controlled validation trials for relatively small patient numbers, and the challenge of appropriately designing the therapy according to genomic classifiers. Moreover, as in many other profiling tasks, there is a lack of concordance between prognostic gene expression signatures for Neuroblastoma derived from different methods and different datasets [24, 25]. In summary, different methods or different datasets genomic classification-induced treatment and personalization on the outcome of high risk Neuroblastoma patients is still an open issue. We present here a novel multi-objective deep learning [26] solution named CDRP (Concatenated Diagnostic Relapse Prognostic) that combines both prognostic and diagnostic information from high-throughput gene expression data. We apply the CDRP architecture to improve classification of high risk patients in two major Neuroblastoma cohorts, showing that as a useful byproduct the training defines an embedding transformation that characterizes better survival analysis.

This is not the first attempt to employ neural networks in Neuroblastoma: a multilayer perceptron has been used to predict Neuroblastoma from expression data in a shallow learning setting [27]. Deep learning has also been proposed for Neuroblastoma, but using bioimages as inputs [28].

The CDRP architecture is built in multiple steps. We train on half of the patients a multitask net (CDRP-N) for classification over two distinct prognostic tasks censoring at 5-years, namely Event-Free Survival (EFS: events are relapse, disease progression or death), and Overall Survival (OS: partitioning patients as either dead or alive). Furthermore, the shared layer of the multitask net uses additional inputs from an autoencoder network (CDRP-A) that models the High-Risk (HR) endpoint, defined as high risk versus non high risk status. The key point is that we train on different tasks the two components over the same data, linking CDRP-A to CDRP-N through an embedding. In order to control for selection bias, both the net CDRP-N and the autoencoder CDRP-A are trained and evaluated using a Data Analysis Protocol (DAP), based on a 10 × 5-fold cross validation developed within the MAQC-II and SEQC studies led by the US FDA [1, 29].

We validate CDRP on the SEQC-NB collection of the RNA sequencing (RNA-Seq) samples from the SEQC study [29, 30]; further, we replicate the analysis on TARGET-NB, a dataset that includes array expression profiles from the TARGET project [31, 32]. To maintain comparability with published results, for the SEQC-NB we adopted the same dataset split employed in the Neuroblastoma SEQC satellite study [30]. On both SEQC-NB and TARGET-NB, we compared CDRP with machine learning algorithms known to perform well on omics data such as Random Forest (RF) and (linear) Support Vector Machines (LSVMs), using the Matthews Correlation Coefficient (MCC) as evaluation metric. Overall, the CDRP architecture consistently achieves same or higher MCC than RF and LSVM on all tasks, with a relevant improvement on published results on the harder task of predicting survival on high risk patients: for instance, CDRP has MCC = 0.38 on SEQC-NB EFS restricted to HR patients versus MCC = 0.21 reached by LSVM. In the paper, we also analyze the model for interpretability: we show that one layer of the CDRP-N can be used to define a new feature space where the SEQC-NB data are naturally ranked for disease severity on a manifold. Further, the embedding can be used to derive an improved survival analysis, detecting a group of Neuroblastoma patients of intermediate risk. We expect that this approach can be tailored for similar prognostic tasks and other malignancies, where patients are screened by clinical-pathological algorithms [33], such as breast cancer [34]. Our approach makes it possible to include in a model, as a part of the neural architecture, an established clinical algorithm already adopted by the scientific community, and put into practice after relevant consensus and approval processes have been achieved.

## Materials and methods

### Data description

The first dataset used in this study (“SEQC-NB”) collects RNA-Seq gene expression profiles of 498 Neuroblastoma patients, published as part of the SEQC initiative [29, 30]. The following endpoints are considered for classification tasks:

- the occurrence of an event (progression, relapse or death) (Event-Free survival, “EFS”);
- the occurrence of death from disease (Overall Survival, “OS”);
- the occurrence of an event (“EFS_HR_”) in High-Risk (HR) patients only;
- the occurrence of death from disease (“OS_HR_”) in HR patients only.

HR status was defined according to the NB2004 risk stratification criteria [35]. The samples were split into training (NBt) and validation (NBv) sets following a published partitioning [30]. Stratification statistics for NBt and NBv are reported in Table 1. RNA-Seq data were preprocessed as *log*_2_ normalized expressions for 60, 778 genes (“MAV-G”) [30]. Expression tables were filtered before downstream analyses by removing features without EntrezID and with interquartile range (IQR) larger than 0.5 using the *nsFilter* function in the *genefilter* R package, leaving 12, 464 (20.5%) genes for downstream analysis. Feature filtering was performed on NBt data set and applied on both NBt and NBv sets to avoid information leakage.

**Table 1:** Sample stratification (left) and summary statistics (right) for the NBt and NBv subset for the covariates High-Risk (HR), Overall Survival (OS) and Event-Free Survival (EFS). HR 0:non high risk, 1:high risk, EFS 0:no event, 1:event, OS 0:alive, 1:dead.

HR	EFS	OS	NBt	NBv
0	0	0	129	130
	1	0	26	24
		1	8	5
1	0	0	31	25
	1	0	12	16
		1	43	49

The second dataset (“TARGET-NB”), originally described in [31], includes Affymetrix Human Exon Array expression profiles of 17, 450 genes for 247 primary diagnostic Neuroblastoma specimens from the TARGET NBL cohort. Classification endpoints are the same used for SEQC-NB, *i*.*e*., EFS, OS, EFS_HR_ and OS_HR_. The dataset was split into training (TGt, *n* = 123) and validation subsets (TGv, *n* = 124) using the train_test_split function of the Python module scikit-learn [36], setting the seed of the pseudorandom number generator to 70. This particular split TGt/TGv was chosen out of 100 random train/test splits as the one where a (linear) Support Vector Machine (LSVM) model reached the best compromise between performance and smaller overfitting effect, measured as the difference between performance on validation and performance on training. The collection of Jupyter notebooks reporting gathered statistics on the TARGET-NB dataset, along with plots and the code used to generate the 100 train/test splits are available on GitLab at the address https://gitlab.fbk.eu/MPBA/CDRP/tree/master/notebooks/target-dataset. As a performance metric, we use the Matthews Correlation Coefficient (MCC) [37–39], which in the binary case reads as $\text{MCC}=\frac{\text{TP}\cdot \text{TN}-\text{FP}\cdot \text{FN}}{\sqrt{\left(\text{TP}+\text{FP})(\text{TP}+\text{FN})(\text{TN}+\text{FP})(\text{TN}+\text{FN}\right)}}$, for TN, TP, FN, FP the entries of the binary confusion matrix.

The sample distribution for the different endpoints is summarized in Table 2. The cohort is highly imbalanced: 83.2% samples in this dataset belong to the HR class.

**Table 2:** Sample stratification (left) and summary statistics (right) for the TARGET-NB TGt and TGv subset for the covariates High-Risk (HR; 0: Non high risk; 1: High risk), Overall Survival (OS; 0: Alive; 1: Dead) and Event-Free Survival (EFS; 0: No event / censored; 1: Event).

HR	EFS	OS	TGt	TGv
0	0	0	15	15
	1	0	0	0
		1	0	0
1	0	0	28	33
	1	0	7	9
		1	73	67

For both SEQC-NB and TARGET-NB datasets, all the available clinical features for each patient (EFS, OS, HR, INSS for TARGET-NB and the additional Age, Gender, Country and Clinical Outcome for SEQC-NB) are detailed in S1 Table.

### Structure of CDRP

**Fig 1:**
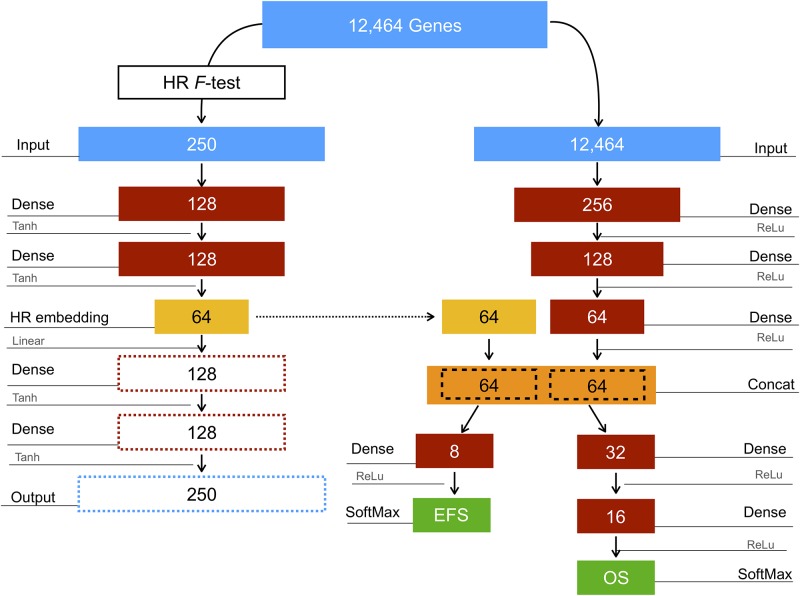
Deep learning architecture. The layer/node structure of the CDRP deep learning architecture. On the left side: the CDRP-A autoencoder; on the right side: the CDRP-N component, with two branches. Blocks indicate net layers, with the input dimensions for the SEQC-NB dataset.

The CDRP architecture, composed of two deep learning network models, referred to as CDRP-A and CDRP-N, is shown in Fig 1. The CDRP-A autoencoder is composed by two specular models, namely *encoder* and *decoder*, designed to learn a representation of the HR/non-HR signal by minimizing the mean squared reconstruction error *mse*). The *encoder* network is composed of an initial input layer of 250 nodes, corresponding to the 2% of the total number of features, as resulting from the DAP ANOVA F-score selection algorithm, with *mse* = 0.042 (CI: (0.041;0.043)). Two fully-connected (dense) layers (128 nodes and tanh activations) and an encoding layer (64 nodes and linear activation) complete the structure of the network. The output of the encoding layer is later used as the *HR embedding* input for the shared merge layer in CDRP-N, while the specular decoding network structure (dotted boxes and arrows in Fig 1) is not used. CDRP-N is a multi-task deep network composed by a shared top structure, and two specialized branches for the two classification tasks considered, namely EFS and OS. The top structure is composed by an initial input layer of dimension 12,464 as the whole set of features, followed by three fully connected layers with 256, 128, and 64 nodes, respectively. The parameters of these layers are shared between the two classification tasks, so that a joint representation can be learned during the training process. The output of the last dense layer is then concatenated with the *HR embedding* layer as computed by CDRP-A. Up to this layer, all activations are ReLU functions [40, 41], with neither dropout [42] nor batch normalization [43]. The network branch for the EFS task consists of a single dense layer with 8 nodes with ReLu activation, followed by a classification SoftMax layer. The branch for the OS task has two dense layers, with 32 and 16 nodes, respectively, and a final decision layer with SoftMax activation. The categorical cross-entropy loss function is used for both tasks, in combination with the Adadelta optimization algorithm [44], with *δ* = 0 and *η* = 1. Two different loss weights coefficients have been empirically assigned to the EFS and the OS tasks, namely 1.0, and 2.0, respectively: the loss value minimized by the network corresponds to the weighted sum of all the individual losses. All hyper-parameters, as well as the final network architecture, have been empirically chosen after a grid search over multiple DAP experiments. The training process of the CDRP-N has batch size 64, in combination with a class weight strategy to cope with unbalanced samples in batches. The number of epochs is bounded to 500, with an early stopping rule on the validation loss, with patience = 4 and min_Δ_ = 10^−6^. The CDRP-A has been trained using the RMSProp [45] optimizer combined with the mean squared error loss function, 2,000 epochs with no stopping criterion, and batch size 64. CDRP is implemented in the Keras [46] framework with TensorFlow [47] backend. All the experiments have been conducted on nVidia Pascal-GPU blades equipped with two GTX 1080, 8GB dedicated RAM, 2,560 CUDA cores, up to 9TFlops throughput and 8 CPU Intel Core i7-6700 with 32 GB RAM. The source code is publicly available in the Git repository https://gitlab.fbk.eu/MPBA/CDRP/.

### The analysis pipeline

**Fig 2:**
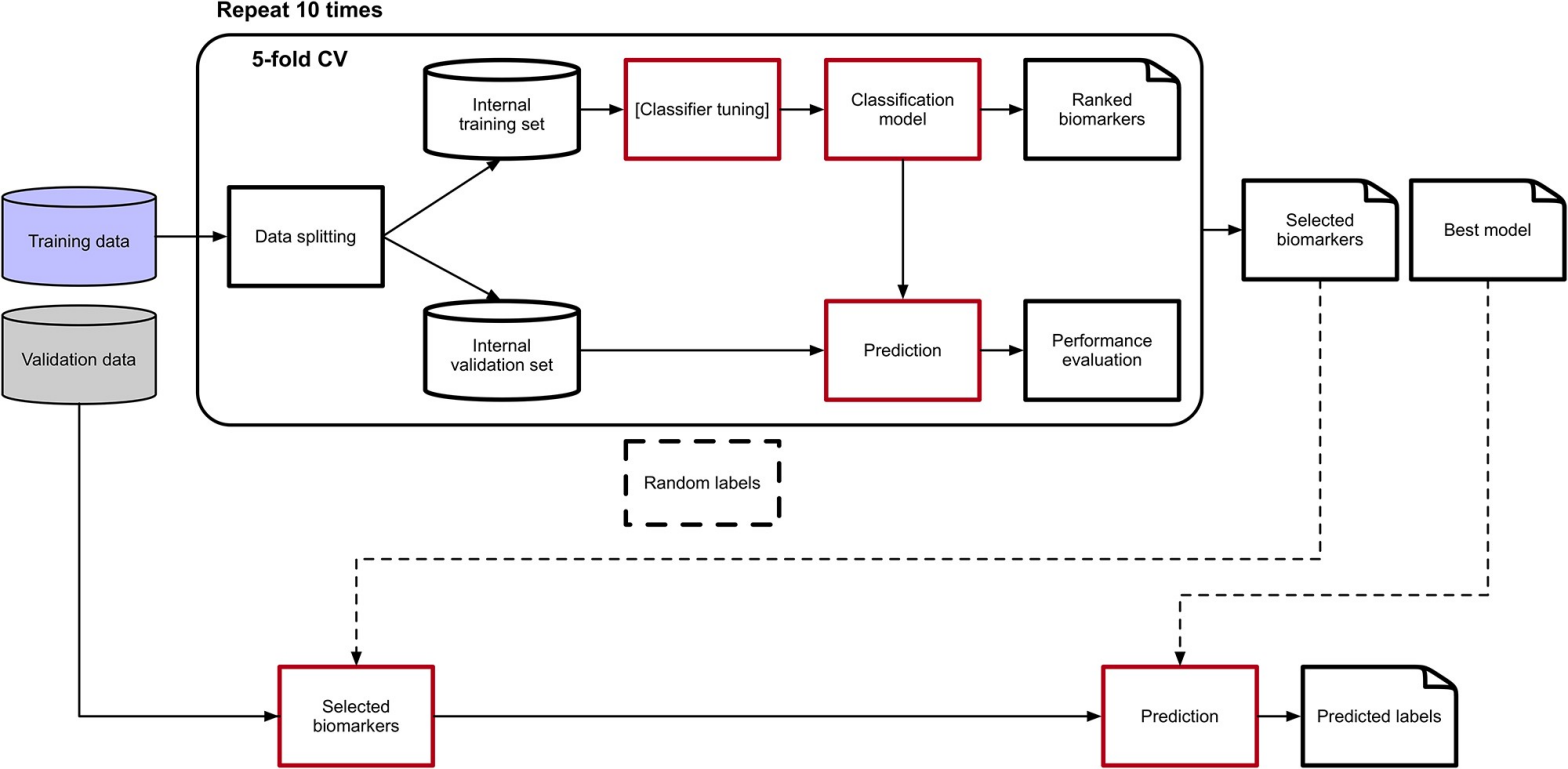
Machine learning analysis pipeline. The Data Analysis Protocol (DAP) used in the experiments, originally defined in the US-FDA MAQC-II initiative.

The experimental methodology is outlined in Fig 2 and follows the Data Analysis Protocol (DAP) developed in the context of the MAQC-II challenge [1], the U.S. Food and Drug Administration (US-FDA) initiative aimed to establish reproducibility in microarray gene expression experiments. Given a dataset divided in a training and a test set, the former undergoes a 10 × 5−fold Stratified Cross Validation [48] resulting in a ranked list of features and a classification performance, measured by MCC. Data are standardized to mean zero and variance one and log_2_ transformed before undergoing classification, and in order to avoid information leakage standardization parameters from the training set are used for both training and test subsets. The *k*-best algorithm [48] is chosen as the feature ranker, CDRP is the classifier and the best model is later retrained on the whole training set and selected for validation on the test set. Furthermore, as a sanity check to avoid unwanted selection bias effects, the pipeline is repeated 20 times with two randomized strategies: a Random Label scheme where the true training labels are stochastically scrambled, and a Random Feature scheme, where a random set of features is selected instead of the optimal list.

### Hidden layer embedding and survival analysis

To investigate the association with the prognosis of the deep features extracted by the activations of different CDRP-N inner layers (including the shared layer), we clustered their deep features by an agglomerative hierarchical algorithm, with Ward linkage and correlation function 1 − (Spearman correlation) as the dissimilarity measure to attribute patients’ labels. The dendrogram was cut so to obtain *k* = 3 clusters. The Kaplan-Meier method was used for estimating overall survival curves, where the cluster labels were used to stratify patients. The log-rank test as implemented in the *survival* R package was used to compare OS between different patients strata. Survival analysis was repeated reweighting samples by inverse probability weighting [49], to take into account the effect of potential clinical confounders. For both SEQC-NB and TARGET-NB, the analysis was adjusted for patient gender; for SEQC-NB, the analysis was also adjusted for country and age of patients, as they were provided among the clinical variables. The distribution of the deep features was further studied with a recent dimensionality reduction algorithm, the Uniform Manifold Approximation and Projection (UMAP) [50]. UMAP searches for local manifold approximations and constructs a topological representation of the high dimensional data into a low dimensional space, minimizing the cross-entropy between the two representations. We used the UMAP implementation in the homonymous R library *umap* (https://github.com/tkonopka/umap), with L2 as the distance metric.

## Results

Results obtained with CDRP solution on the SEQC-NB, and the TARGET-NB datasets are reported in details in Table 3, and in Table 4, respectively. Results obtained by other machine learning models are also reported for comparison, namely (linear) Support Vector Machine (LSVM), Random Forest (RF), CDRP-N network (no autoencoder contribution).

**Table 3:** Comparison of the median MCC from the SEQC-NB study in cross-validation (“NBt”) and external validation (“NBv”) with the MCC obtained by CDRP. For LSVM, RF, CDRP-N and CDRP-A+CDRP-N, 95% studentized bootstrap confidence intervals for NBt are also reported.

Task	SEQC		LSVM		RF		CDRP-N		CDRP-A+CDRP-N	
	NBt	NBv	NBt	NBv	NBt	NBv	NBt	NBv	NBt	NBv
EFS	0.45	0.50	0.46 (0.43;0.49)	0.48	0.45 (0.41;0.48)	0.52	0.40 (0.36;0.45)	0.41	0.42 (0.38;0.45)	0.45
OS	0.48	0.47	0.46 (0.42;0.50)	0.47	0.43 (0.39;0.47)	0.37	0.48 (0.46;0.53)	0.48	0.50 (0.45;0.54)	0.57
EFS_HR_	0.34	0.16	0.13 (0.08;0.18)	0.21	0.17 (0.10;0.23)	0.13	0.15 (0.09;0.22)	0.19	0.18 (0.11;0.25)	0.38
OS_HR_	0.36	0.07	0.22 (0.16;0.28)	0.12	0.33 (0.26;0.39)	0.10	0.23 (0.21;0.35)	0.14	0.25 (0.19;0.31)	0.19

**Table 4:** Comparison of the median MCC from the TARGET-NB dataset in cross-validation (“TGt”) and external validation (“TGv”) with the MCC obtained by CDRP. 95% studentized bootstrap confidence intervals for TGt are also reported.

Task	LSVM		RF		CDRP-N		CDRP-A+CDRP-N	
	TGt	TGv	TGt	TGv	TGt	TGv	TGt	TGv
EFS	0.40 (0.34;0.45)	0.40	0.35 (0.29;0.41)	0.22	0.36 (0.30;0.42)	0.25	0.38 (0.33;0.44)	0.43
OS	0.41 (0.36;0.46)	0.42	0.28 (0.23;0.33)	0.35	0.31 (0.25;0.37)	0.24	0.34 (0.30;0.40)	0.39
EFS_HR_	0.12 (0.05;0.19)	-0.01	0.07 (0.02;0.13)	0.12	0.16 (0.08;0.24)	0.08	0.17 (0.09;0.24)	0.18
OS_HR_	0.14 (0.08;0.20)	0.12	-0.02 (-0.04;-0.01)	0.01	0.19 (0.13;0.26)	0.07	0.21 (0.11;0.27)	0.27

Although no clear advantage is provided on the training portion of SEQC-NB, CDRP improves MCC in validation for the OS endpoint, and to our knowledge it is the first model to improve on the High-Risk cohort (EFS_HR_, OS_HR_). Furthermore, considering results obtained on the TARGET-NB dataset, the two architectures CDRP-N and CDRP-A+CDRP-N are confirmed as the best performing in cross-validation on TGt for the HR tasks, with CDRP-A+CDRP-N outperforming LSVM, RF and CDRP-N on TGv. Notably, the very same architecture used for the SEQC-NB dataset has been applied on the TARGET-NB with no hyper-parameter tuning nor further customizations. This demonstrates the validity of the proposed CDRP solution on being able to distill the diagnostic algorithm, which represents a crucial boosting on the learning process of the prognostic predictions. Obtained results are encouraging to look for further improvements, especially related to the interpretability of features synthesized by the network. A theoretical basis justifying the achieved improvement relies on the fact that the information distilled from the diagnostic task adds clinical information, used by the multi-task predictor, which combines the OS and EFS tasks.

**Fig 3:**
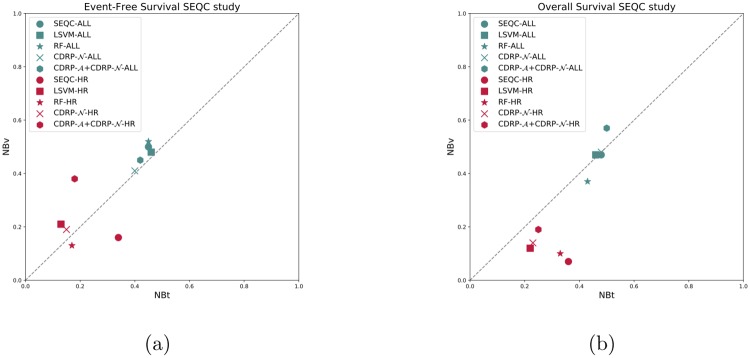
Comparison of cross-validation vs validation performance on the SEQC-NB dataset. (a) Event-free survival classification task; (b) Overall survival classification task.

**Fig 4:**
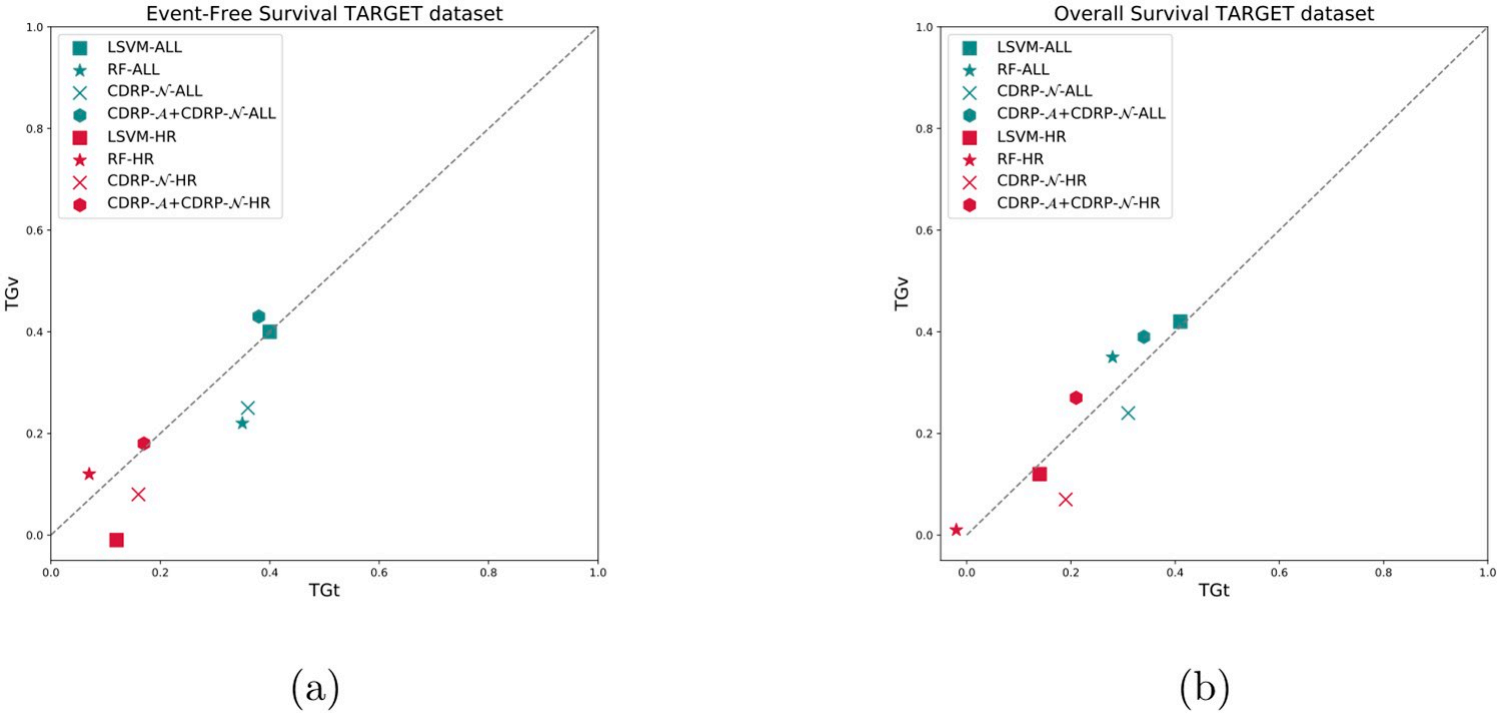
Comparison of cross-validation vs validation performance on the TARGET-NB dataset. (a) Event-free survival classification task; (b) Overall survival classification task.

**Fig 5:**
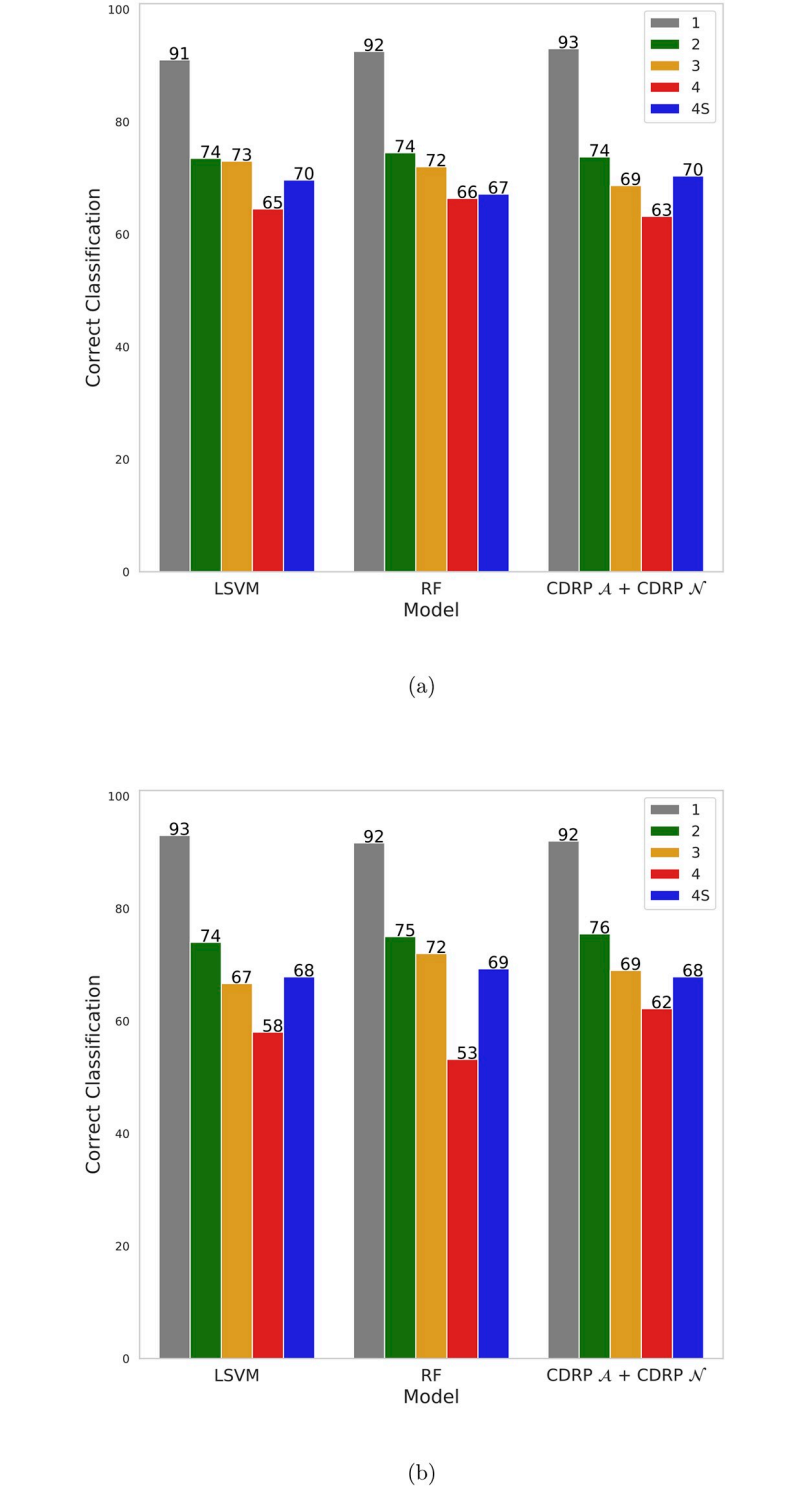
Percentage of correct classification in DAP training by different models stratified for INSS stage for (a) SEQC-NB EFS (b) SEQC-NB OS.

**Fig 6:**
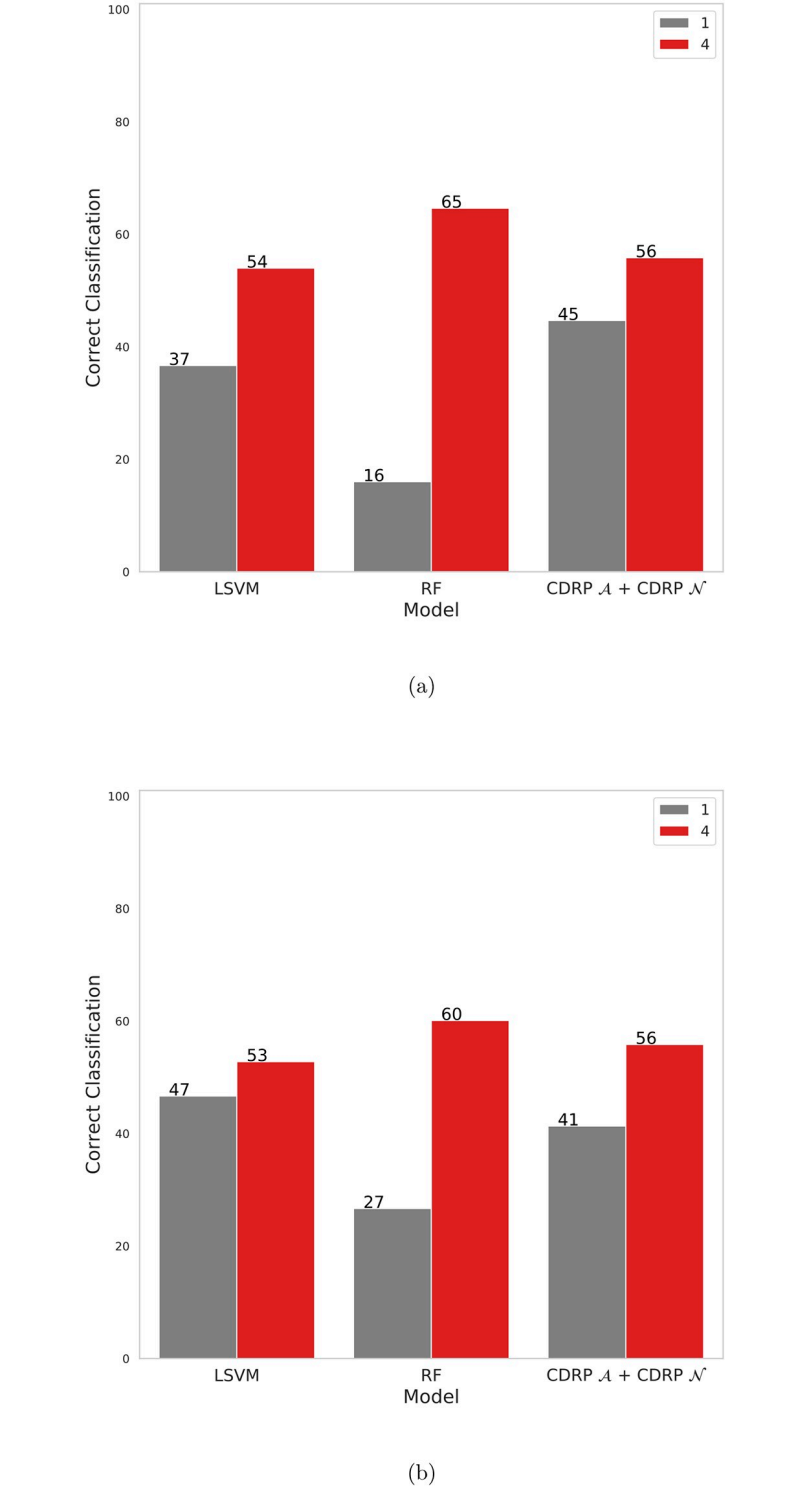
Percentage of correct classification in DAP training by different models stratified for INSS stage for (a) TARGET-NB EFS (b) TARGET-NB OS.

**Fig 7:**
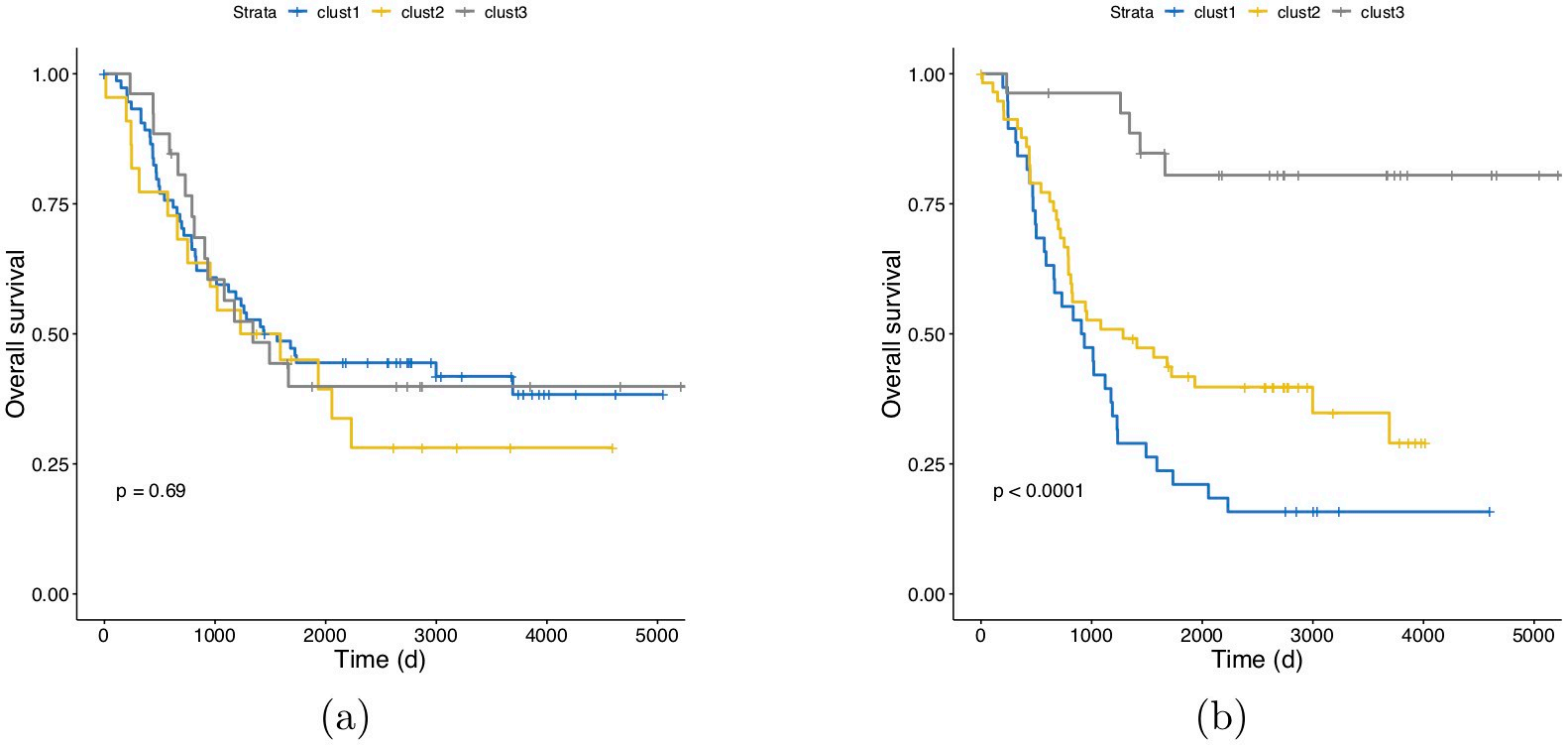
Kaplan-Meier overall survival analysis on TARGET-NBt. (a) Patient stratification defined by hierarchical clustering based on the deep features extracted from the 64-node shared layer of CDRP, without the contribution of CDRP-A; (b) Patient stratification defined by hierarchical clustering based on the deep features extracted from the 128-node merged layer of CDRP, with the information distilled from the CDRP-A diagnostic task. p: log-rank p-value.

**Fig 8:**
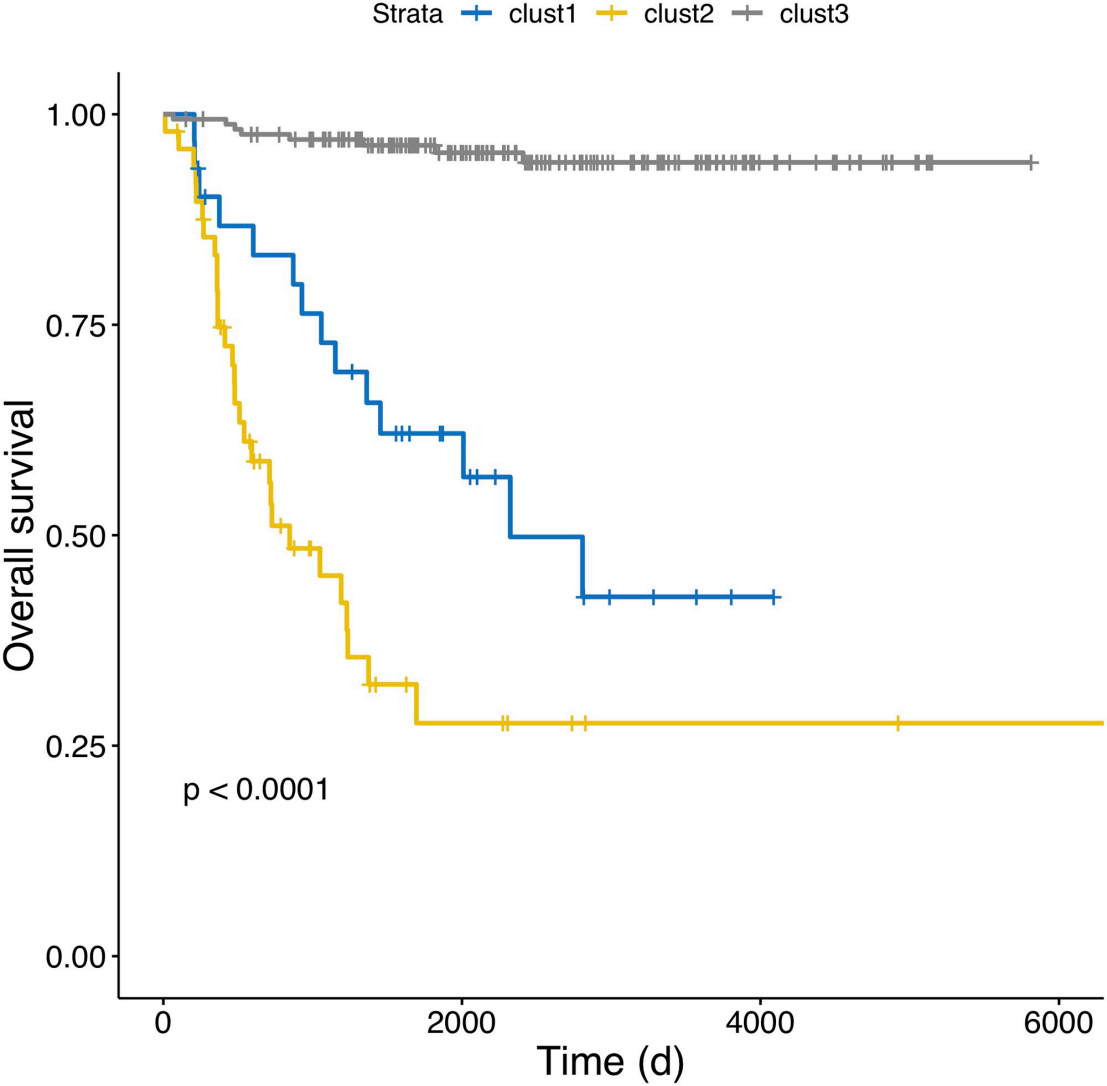
Kaplan-Meier overall survival analysis on SEQC-NBt. Patient stratification was defined by hierarchical clustering based on the deep features extracted from the 32-node OS branch of CDRP (see Fig 1). p: log-rank p-value.

**Fig 9:**
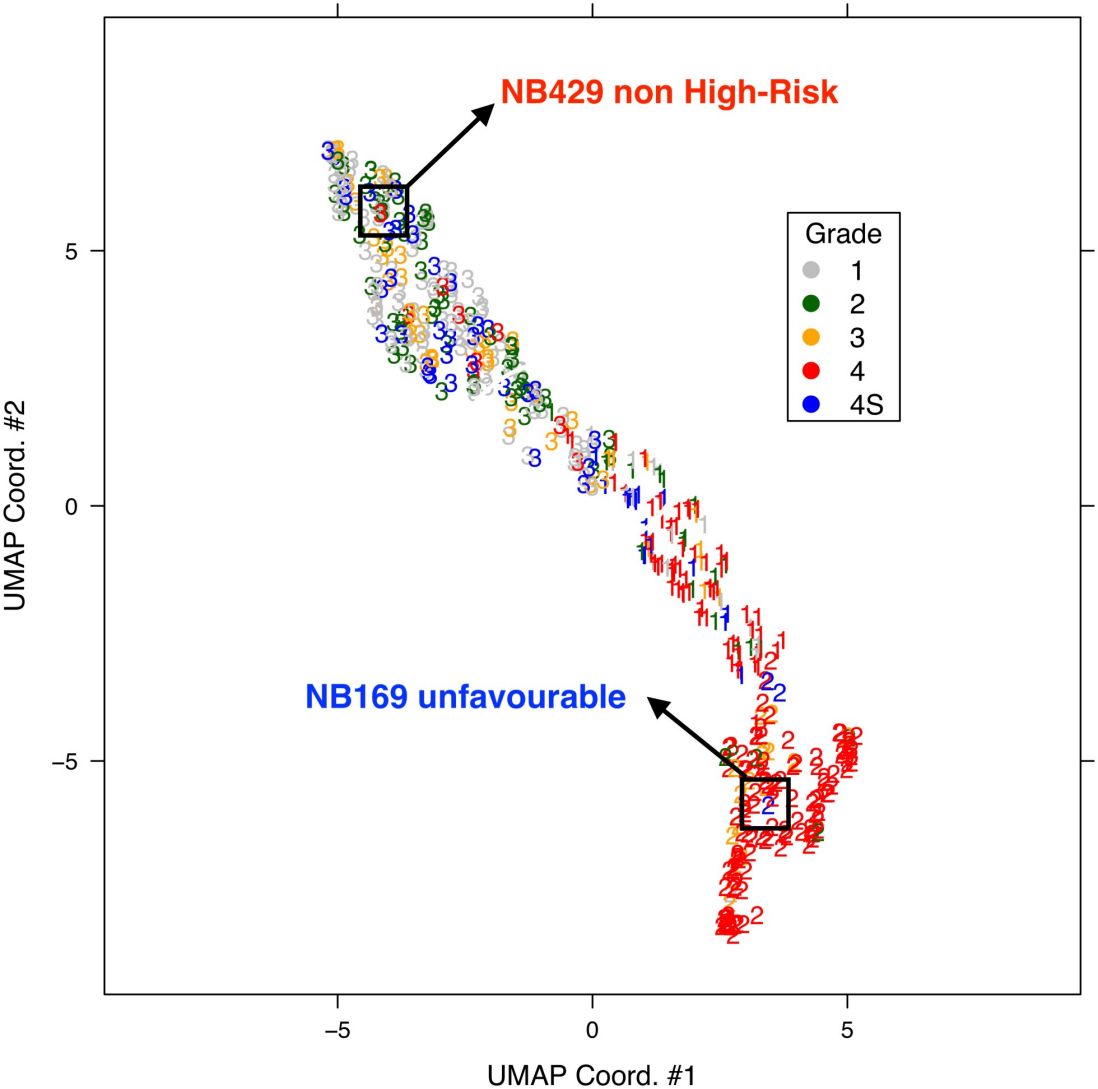
UMAP projection of the 1000 deep features of SEQC-NBt samples on the hidden Overall Survival layer with 32 nodes. Colors indicate tumor grade, while numbers correspond to the hierarchical clusters of Fig 8. Two outlier samples are highlighted.

CDRP models with random labels yield MCC ≈ 0, indicating honest estimates, while consistent results are obtained also with swapped training and validation sets. A plot comparing the performance of the CDRP solution and other machine learning models is reported in Fig 3 for the SEQC-NB dataset, and in Fig 4 for the TARGET-NB dataset. In particular, these plots show results obtained on internal validation (*x*-axis) and external validation sets (*y*-axis) for the EFS and OS tasks on the entire patients cohort (in green), and the EFS_HR_ and OS_HR_ tasks on the HR cohort (in red). For SEQC-NB, a consistent correlation emerges between classifiers’ performance and INSS stage, as shown in Fig 5, reporting the percentage of correct classification during the DAP training: samples with INSS stage 1 are better classified than samples in different stages, with a decreasing trend for increasing disease severity; samples with INSS 4 result the hardest to classify. Notably, this does not hold in the TARGET-NB dataset, where samples with INSS 4 are consistently better classified than samples with INSS 1, as displayed in Fig 6. In S1–S12 Figs the classification results are detailed for each samples across the 10 replicates of the 5-fold Cross Validation schema. Using the 64 TARGET-NB deep features extracted after the activation of the shared layer of CDRP-N (“shared_64”) to cluster TGt patients, we observe no significantly different OS curves among patient strata (Fig 7, panel a). Remarkably, using the 128 TARGET-NB deep features from the shared merged layer of CDRP-A+CDRP-N (“merge_128”), the TGt patients stratify into groups with significantly different OS (log-rank *p* < 10^−4^, Fig 7, panel b). The survival analysis was also adjusted for patient gender by inverse probability weighting, with unchanged results (see S13 Fig). A full description of the clusters’ stratification for INSS stage, risk and binary survival endpoint is provided in Table 5. We also tested the CDRP embeddings for patient subtypes by considering the structure of the dendrogram resulting from the unsupervised hierarchical clustering of SEQC-NBt. We divided patients into three groups using the SEQC-NB deep features extracted in correspondence of the 32-node Dense layer of CDRP (see Fig 1) and identified a novel patient stratification in three subtypes with significantly different overall survival curves (log-rank *p* < 10^−4^, Fig 8). Adjusting for clinical confounders did not highlight any impact on survival (see S13 Fig). The same three clusters (1:gray, 2: yellow, 3:blue) are mapped in the UMAP planar projection of the same data displayed in Fig 9, where the point label indicates cluster membership, while color denotes patient INSS grading.

**Table 5:** Distribution of patients in the 3 hierarchical clusters stratified by INSS stage, risk and binary survival endpoint.

Dataset	Split	Cluster	EFS (0/1)	OS (0/1)	HR (Y/N)	INSS (1/2/3/4/4S)
SEQC-NB	NBt	1	1/14	7/8	11/4	0/0/1/14/0
		2	21/42	27/36	58/5	3/2/6/50/2
		3	138/33	164/7	17/154	57/38/23/27/26
	NBv	1	36/28	53/11	22/42	10/9/6/31/8
		2	21/52	31/42	67/6	0/6/11/55/1
		3	98/14	111/1	1/111	51/23/16/6/16
	All	1	33/38	55/16	31/40	8/10/4/39/10
		2	36/97	52/75	119/8	1/5/17/102/2
		3	246/54	286/14	26/274	112/63/42/42/41
TARGET-NB	TGt	1	3/35	6/32	0/38	0/0/0/38/0
		2	21/37	22/36	1/57	1/0/0/57/0
		3	19/8	22/5	14/13	14/0/0/13/0
	TGv	1	23/56	27/52	0/79	1/0/0/78/0
		2	23/10	26/7	14/19	14/0/0/19/0
		3	2/10	4/8	1/11	1/0/0/11/0
	ALL	1	26/52	30/48	0/78	1/0/0/77/0
		2	34/10	36/8	28/16	28/0/0/16/0
		3	31/94	41/84	2/123	2/0/0/123/0

**Fig 10:**
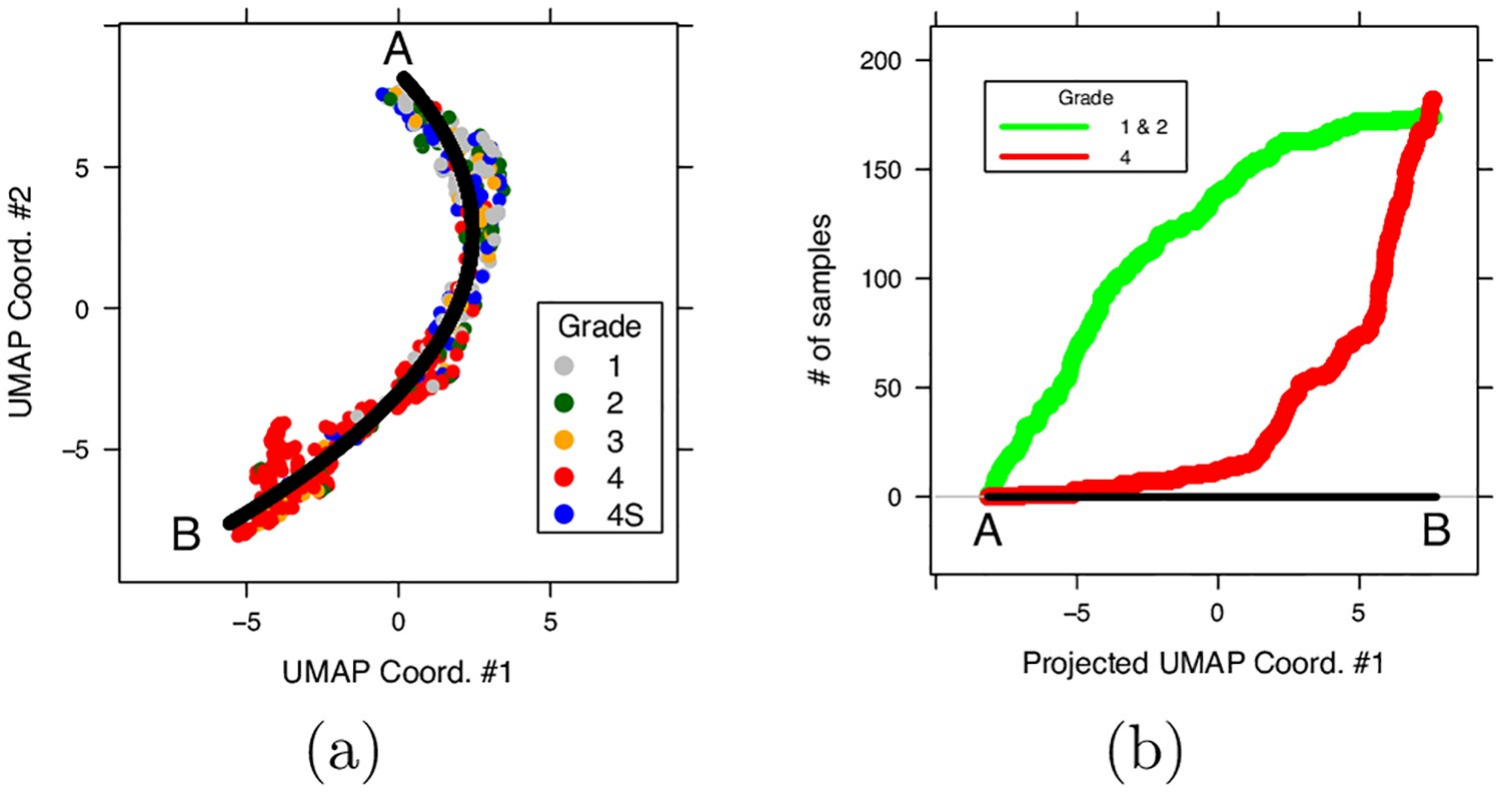
Manifold approximation of UMAP projection. (a) Colors indicate tumor grade and the black line is the approximating parabola; (b) Cumulative sum of severe (red line) and less severe (green) cases while traversing the linearly projected manifold from point A to point B. Samples with low grading and favorable prognosis concentrate close to point A, while patients with more severe condition or unfavorable prognosis are grouping towards point B.

Notably, severity progression of the three clusters is modeled by the UMAP dimensionality reduction algorithm. The resulting manifold can be effectively approximated by the parabola *x* = −1.896671 + 0.403570*y* + 0.075521*y*^2^, which results the best curve among all conics in term of min square error (Fig 10, panel a). If the manifold is traversed from top left (point A in the figure) to bottom right (point B), and the samples projected of the fitting parabola, there is a growing trend of samples with bad prognosis. This is also highlighted by the different INSS grading of the samples, with patients of grade 4 accumulating towards the lower portion of the manifold (Fig 10, panel b). It is also worth noting that the network embedding correctly locate two interesting outliers (highlighted in Fig 9):

1. Sample NB249, a patient that, despite being INSS stage 4, is a non-High-Risk case; the corresponding point is indeed projected on the top left portion of the manifold together with all the less severe cases; this sample is always correctly classifies by CDRP, as shown by S1–S12 Figs.
2. Sample NB169, a grade 1 patient who nonetheless had an unfavorable prognosis; on the projected manifold, its blue “2” mark can be correctly found in the bottom right zone populated by the most severe grade 4 patients; this sample is always misclassified in training by CDRP for the EFS task, and correctly classified only in 4 replicates out of 10 for the OS task.

## Conclusion

CDRP is a novel multitask deep learning architecture that improves prediction of hard prognostic endpoints by injecting latent variables derived by autoencoding a standard clinical model. The approach leverages the advent of deep learning structures that can flexibly integrate multimodal data or create internally multiple processing paths. In this study, the autoencoder component clearly improves prediction of survival for high risk patients. Further, the network can be used to generate embeddings associated with disease severity, improving on initial tumor grading.

The DAP adapted from the MAQC experience has been instrumental in avoiding risk of selection bias. Remarkably, more than 11 billion parameters have been trained in total, confirming the need for a rigorous control of the model selection process.

The architecture can be naturally extended with multi-modal inputs by adding appropriate embeddings: in particular embeddings for clinical variables and image data, as well as multi-omics integration are being investigated.

## Supporting information

S1 Table Clinical descriptors of all patients in the SEQC-NB and the TARGET-NB dataset, split in training and test portions. **Sample** the ID of the sample in the original dataset; **HR** the binarized High Risk, 0: low risk, 1: high risk, **EFS** the binarized Event Free Survival, 0: no event / censored, 1: event, **OS** the binarized Overall Survival, 0: alive, 1: dead, **EFS (days)** Event Free Survival in days, **OS (days)** Overall Survival in days, **INSS** Neuroblastoma INSS stage, **Clinical outcome**, favorable / unfavorable, **Age (days)** Age in days, **Gender** M: male, F: female, **Country** patient country.
(XLSX)

S1 Fig Pictogram of the number of times each SEQC-NB sample has been correctly classified during the 10x5-CV DAP training phase by the CDRP-A+CDRP-N model for the EFS task. (PDF)

S2 Fig Pictogram of the number of times each SEQC-NB sample has been correctly classified during the 10x5-CV DAP training phase by the CDRP-A+CDRP-N model for the OS task. (PDF)

S3 Fig Pictogram of the number of times each SEQC-NB sample has been correctly classified during the 10x5-CV DAP training phase by the RF model for the EFS task. (PDF)

S4 Fig Pictogram of the number of times each SEQC-NB sample has been correctly classified during the 10x5-CV DAP training phase by the RF model for the OS task. (PDF)

S5 Fig Pictogram of the number of times each SEQC-NB sample has been correctly classified during the 10x5-CV DAP training phase by the LSVM model for the EFS task. (PDF)

S6 Fig Pictogram of the number of times each SEQC-NB sample has been correctly classified during the 10x5-CV DAP training phase by the LSVM model for the OS task. (PDF)

S7 Fig Pictogram of the number of times each TARGET-NB sample has been correctly classified during the 10x5-CV DAP training phase by the CDRP-A+CDRP-N model for the EFS task. (PDF)

S8 Fig Pictogram of the number of times each TARGET-NB sample has been correctly classified during the 10x5-CV DAP training phase by the CDRP-A+CDRP-N model for the OS task. (PDF)

S9 Fig Pictogram of the number of times each TARGET-NB sample has been correctly classified during the 10x5-CV DAP training phase by the RF model for the EFS task. (PDF)

S10 Fig Pictogram of the number of times each TARGET-NB sample has been correctly classified during the 10x5-CV DAP training phase by the RF model for the OS task. (PDF)

S11 Fig Pictogram of the number of times each TARGET-NB sample has been correctly classified during the 10x5-CV DAP training phase by the LSVM model for the EFS task. (PDF)

S12 Fig Pictogram of the number of times each TARGET-NB sample has been correctly classified during the 10x5-CV DAP training phase by the LSVM model for the OS task. (PDF)

S13 Fig Kaplan-Meier survival analyses with adjustment for clinical confounders. (PDF)
