# Supplementary figures and images for: Distillation of the clinical algorithm improves prognosis by multi-task deep learning in high-risk Neuroblastoma
Source: PLoS One. 2018 Dec 7;13(12):e0208924. doi: 10.1371/journal.pone.0208924 (PMC6285384; doi:10.1371/journal.pone.0208924)

SEQC-NB - EFS - CDRP  $\mathcal{A}$  + CDRP  $\mathcal{N}$

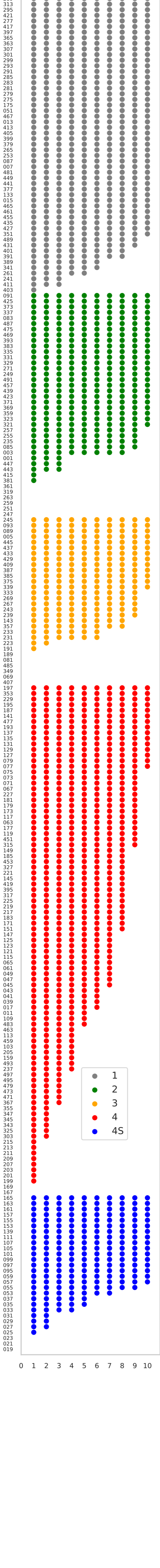

Supplement: S1 Fig — (PDF) [file pone.0208924.s002.pdf]

SEQC-NB - OS - CDRP  $\mathcal{A}$  + CDRP  $\mathcal{N}$

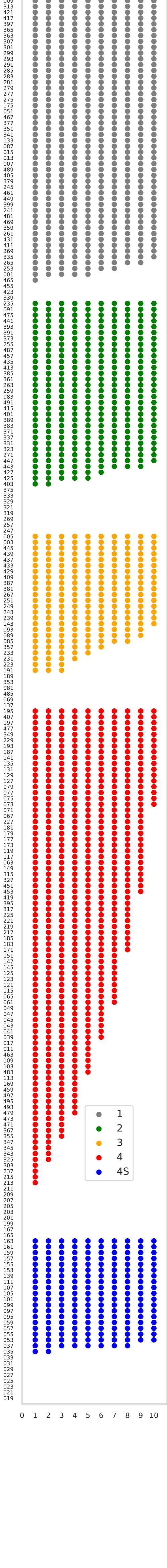

Supplement: S2 Fig — (PDF) [file pone.0208924.s003.pdf]

SEQC-NB - EFS - RF

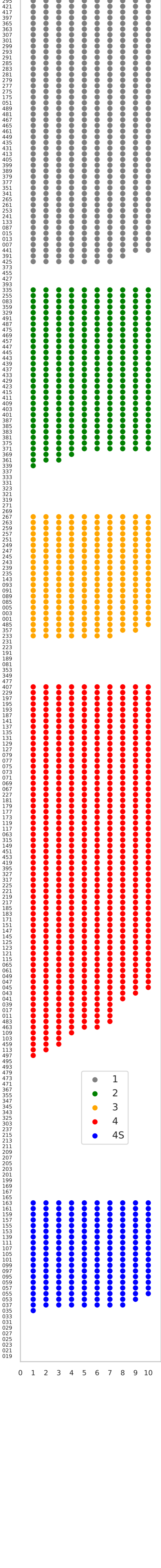

Supplement: S3 Fig — (PDF) [file pone.0208924.s004.pdf]

SEQC-NB - OS - RF

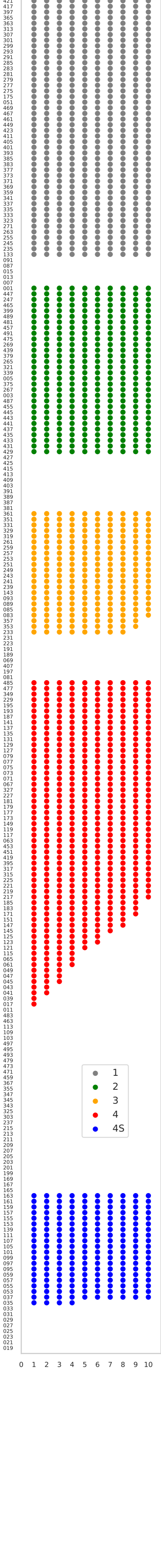

Supplement: S4 Fig — (PDF) [file pone.0208924.s005.pdf]

SEQC-NB - EFS - LSVM

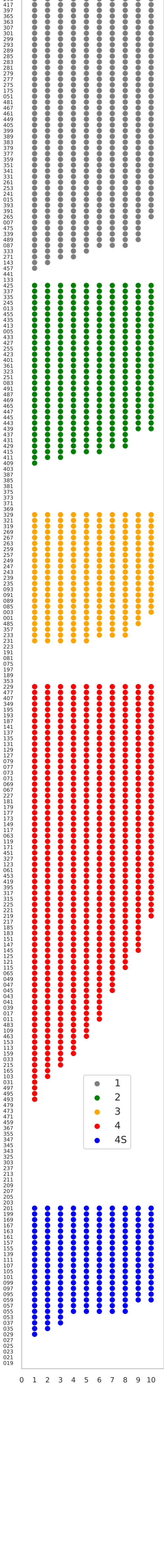

Supplement: S5 Fig — (PDF) [file pone.0208924.s006.pdf]

SEQC-NB - OS - LSVM

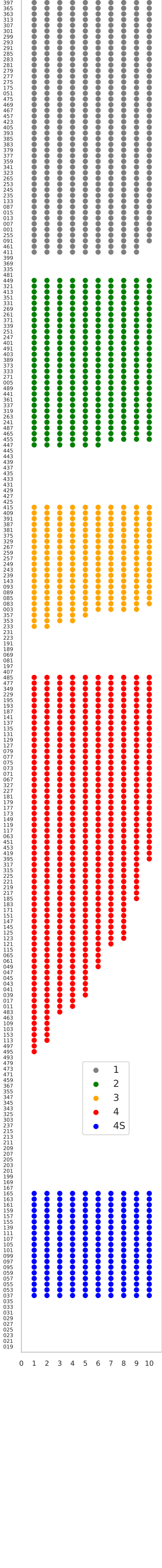

Supplement: S6 Fig — (PDF) [file pone.0208924.s007.pdf]

TARGET - EFS - CDRP  $\mathcal{A}$  + CDRP  $\mathcal{N}$

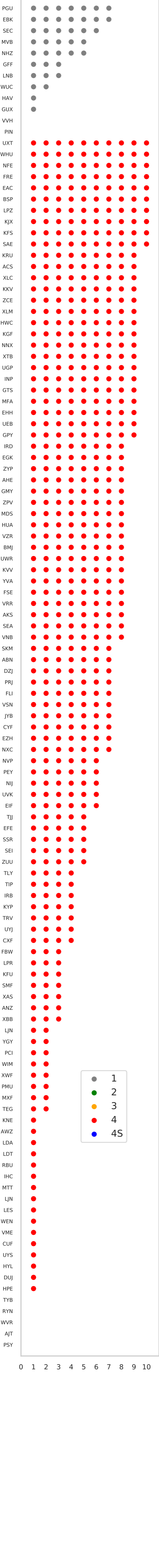

Supplement: S7 Fig — (PDF) [file pone.0208924.s008.pdf]

TARGET - OS - CDRP  $\mathcal{A}$  + CDRP  $\mathcal{N}$

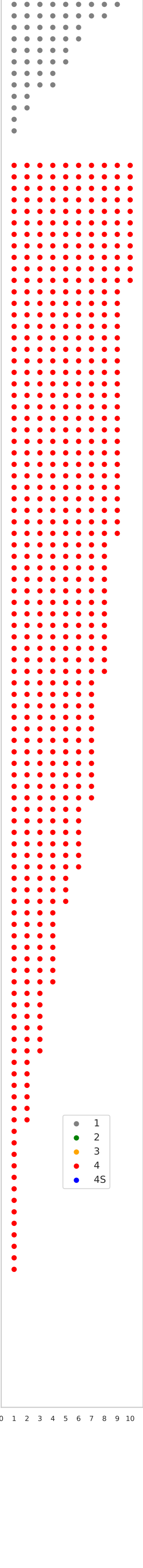

Supplement: S8 Fig — (PDF) [file pone.0208924.s009.pdf]

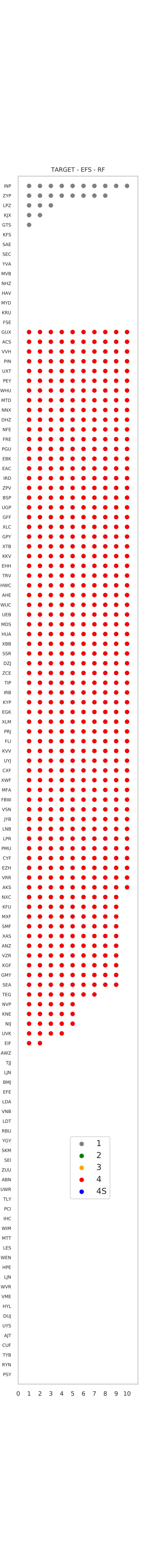

Supplement: S9 Fig — (PDF) [file pone.0208924.s010.pdf]

TARGET - OS - RF

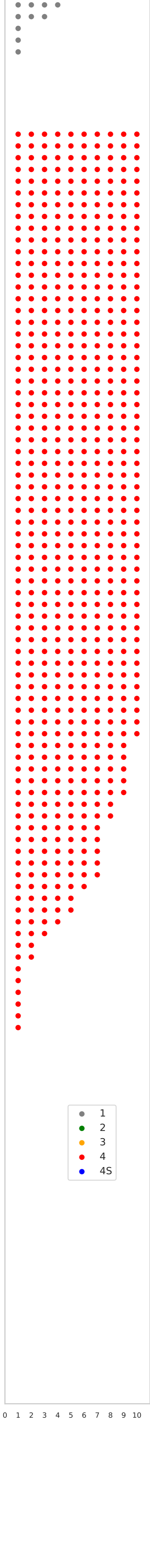

Supplement: S10 Fig — (PDF) [file pone.0208924.s011.pdf]

TARGET - EFS - LSVM

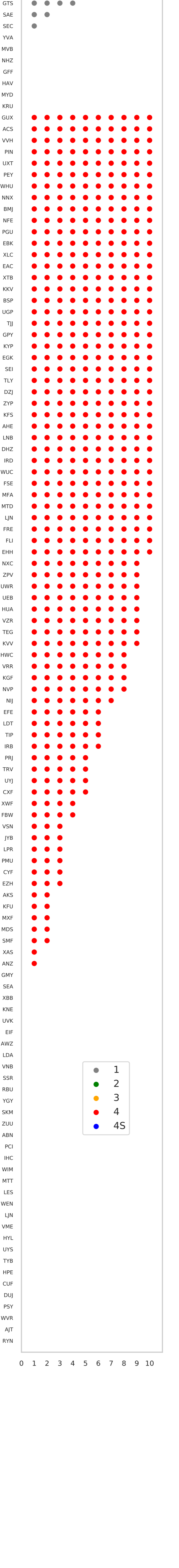

Supplement: S11 Fig — (PDF) [file pone.0208924.s012.pdf]

TARGET - OS - LSVM

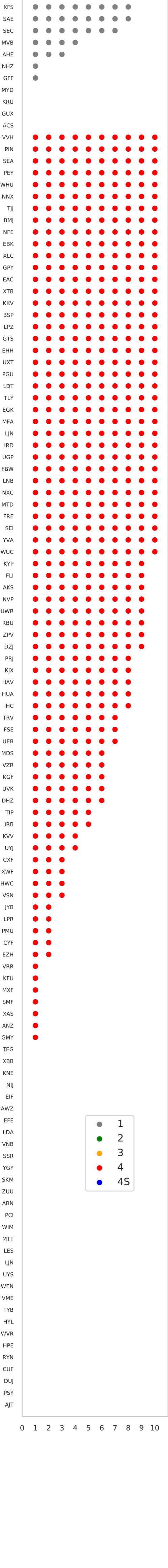

Supplement: S12 Fig — (PDF) [file pone.0208924.s013.pdf]
