# Supplementary material for: Distillation of the clinical algorithm improves prognosis by multi-task deep learning in high-risk Neuroblastoma
Source: PLoS One. 2018 Dec 7;13(12):e0208924. doi: 10.1371/journal.pone.0208924 (PMC6285384; doi:10.1371/journal.pone.0208924)
Supplement: S13 Fig — (PDF) [file pone.0208924.s014.pdf]

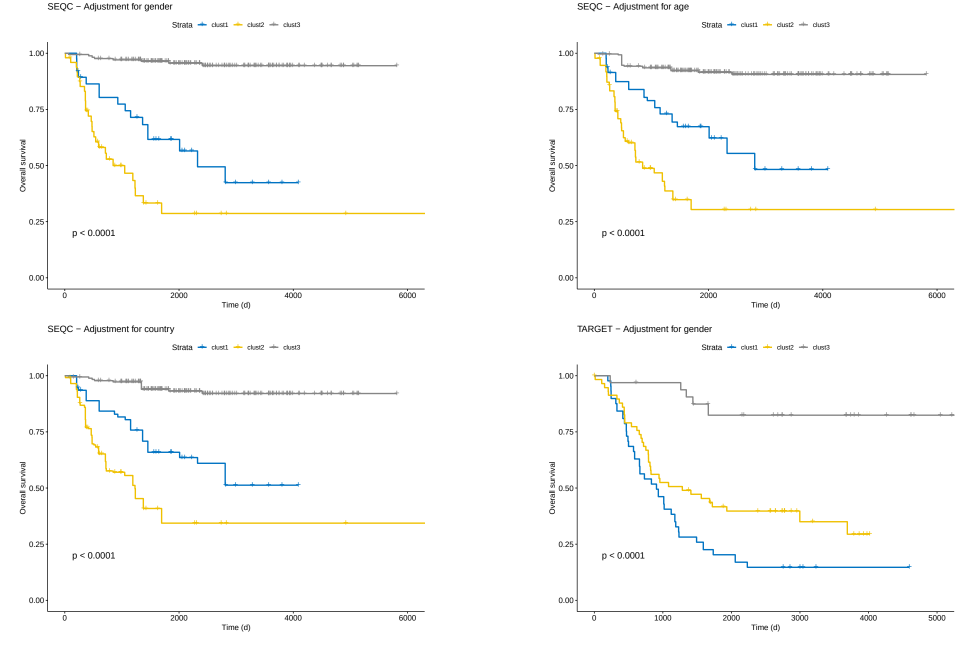
SEQC − Adjustment for gender
Strata clust1 clust2 clust3
Overall survival
Time (d)
p < 0.0001
SEQC − Adjustment for age
Strata clust1 clust2 clust3
Overall survival
Time (d)
p < 0.0001
SEQC − Adjustment for country
Strata clust1 clust2 clust3
Overall survival
Time (d)
p < 0.0001
TARGET − Adjustment for gender
Strata clust1 clust2 clust3
Overall survival
Time (d)
p < 0.0001
